# Continual decision‐making dynamics across biological organisms

**DOI:** 10.1002/brv.70115

**Published:** 2025-12-26

**Authors:** Liberty Severs, Qiuran Wang

**Affiliations:** ^1^ Faculty of Sciences University of Lisbon Campo Grande 016 1749‐016 Lisboa Portugal; ^2^ Institute for Philosophy II Ruhr University Bochum Universitätsstraße 150 44801 Bochum Germany; ^3^ Konrad Lorenz Institute for Evolution and Cognition Research Martinstraße 12 A‐3400 Klosterneuburg Austria

**Keywords:** decision‐making, adaptive behaviour, non‐neural cognition, biological organisation, distributed cognition, slime mould, decentralised system

## Abstract

Decision‐making is a central function of adaptive behaviour in biological agents. However, strategies for adaptive decision‐making can vary substantially across species. Here, we aim to extend the comparative scope of decision‐making analyses to phylogenetically diverse organisms. To do so, we introduce the Continual Decision Making Dynamics (CDMD) framework, which characterises decision‐making as a temporally extended, history‐sensitive process that is sustained by self‐organising and self‐regulating interactions. Drawing on empirical examples, we demonstrate how CDMD can accommodate the organisation of control architectures that support more distributed and decentralised modes of decision‐making, and facilitate a comparative approach to decision‐making strategies across phylogenetic and organisational scales. We discuss how our model can be situated among other related approaches to decision‐making, capturing a distinctive subset of decision strategies that can be modelled in the absence of explicit representational structures. Our framework contributes to integrative approaches that bridge biological complexity and cognitive modelling, and highlights how regulatory control and organisational constraints shape decision‐making dynamics across a broader range of biological systems.

## I. INTRODUCTION

Decision‐making is widely recognised as a hallmark of adaptive behaviour in biological systems (Cisek, 2019, 2022). However, across the cognitive and life sciences, there is substantial divergence in how decision‐making is characterised in relation to cognition and as an empirical target of investigation, making it difficult to assess how such capacities are conserved across taxa and levels of organisation, including within systems traditionally excluded from cognitive analysis. Decision‐making is often defined (in human and non‐human animals) as the selection of an action from among alternative options, typically on the basis of accumulating evidence over time, the integration of information, and evaluative processes (Kahneman & Tversky, 2013; Rangel, Camerer & Montague, 2008; Ratcliff & McKoon, 2008; Shadlen & Kiani, 2013; Simon & Newell, 1971; Usher & McClelland, 2001). Whilst significant progress has been made in understanding the cognitive, computational, and neural mechanisms that support decision‐making along these lines, many influential models assume that the capacity for decision‐making is intrinsically grounded in ‘higher‐order’ processes, including human‐like reasoning and deliberation (Kahneman, 2011; Sloman, 1996), or else restrict measures of decision‐making to discrete choice selection mechanisms in the brain.

More recently, formal models and frameworks such as Bayesian decision‐making (Gershman, Horvitz & Tenenbaum, 2015), reinforcement learning (Sloman, 1996; Sutton & Barto, 1998), and drift‐diffusion (Ratcliff & McKoon, 2008), have been applied in order to tease apart the cognitive computations and psychological processes that support adaptive decision‐making, with extensive evidence of brain‐level mediation by regions such as the prefrontal cortex and basal ganglia circuits (Schultz, Dayan & Montague, 1997; Shenhav, Botvinick & Cohen, 2013). These developments have provided an opportunity to understand better the behavioural dimensions of decision‐making across a variety of contexts, particularly under conditions of uncertainty, by appealing to schemas that structure the process of belief‐updating, reward‐based learning, and/or evidence accumulation in an (approximately) optimal manner (Khoudary, Peters & Bornstein, 2025; Shenhav *et al*., 2013), positing neural representations that mediate decision processes and outcomes. However, there are many contexts in which decision‐making need not be couched in representational terms, and the assumption that only human‐like reasoners are capable of decision‐making has been challenged on several fronts (Barandiaran & Moreno, 2006; Beer, 2021; Huang, Bich & Bechtel, 2021; Levin & Dennett, 2020).

In parallel, normative models derived from frameworks within behavioural ecology and evolutionary biology (e.g. optimal foraging theory, risk‐sensitivity theory) offer a complementary perspective, whereby decision‐making is studied as an interactive, adaptive process that is shaped by environmental factors, including ecological pressures, risks, and contextual demands that collectively structure an agent's learning and decision‐making strategies (Houston & McNamara, 1999; Kacelnik & El Mouden, 2013). Placing more traditional cognitive and computational frameworks *in situ*, these normative perspectives have drawn attention to other relevant factors related to the environment in which an agent makes decisions (and, importantly, the mode in which they do so) as central to our understanding of decision‐making itself.

Whether navigating complex habitats, coordinating collective movement in groups, or selecting between competing foraging options under temporal constraints, organisms are regularly faced with environmental contingencies that require flexible and prompt responses. Here, it has been theorised that many biological organisms – from fish to insects, humans, and everything in between – manage to find adaptive solutions (act/make decisions) that balance energy acquisition with associated risks as a function of state‐dependent trade‐offs, whereby the integration of ecological sources of uncertainty and life‐history constraints modulate decision‐making strategies within a given niche (Houston, McNamara & Hutchinson, 1993; Kacelnik & Cuthill, 1987; McNamara & Houston, 2009; Stephens & Krebs, 1987). Unlike the traditional focus on more deliberative forms of decision‐making as a sequential cognitive process, such approaches focus on the contextual factors and properties of choice behaviour in relation to the organism's evolved niche, and particularly in relation to their fitness‐maximising properties given ecological pressures (Tinbergen, 1963).

Importantly, such an approach often allows for a broader, more contextually relevant notion of decision‐making that is not restricted to the ‘higher‐order’ capacities of humans and other animals (Barnard, 2004). Indeed, there are a number of routes to (and mechanisms for) action selection that enable such strategic choice behaviour, and that are tailored to the constraints of both the environment and agent in order to balance different evolutionary costs and benefits effectively. At the same time, given the range of putative decision strategies and variability in the environment, animals also produce adaptive choice behaviour that appears to resolve certain trade‐offs through decision‐making without the need to *represent* such costs and benefits, instead acting in ways better aligned with the selection of efficient heuristics, biases, and learned response contingencies triggered by specific sensory inputs (Birch, 2024; Brown & Birch, 2025).

This comparative perspective allows us to diversify the model organisms typically associated with decision‐making behaviour, and systematically to interrogate the variety of flexible and inflexible decision rules that can be plausibly applied to resolve certain trade‐offs across deep phylogenetic distances, not just among closely related animals who possess a centralised nervous system. Emerging evidence from non‐neural systems challenges many standard assumptions about decision‐making. Here, decision‐like behaviours can be observed in uni‐ and multicellular organisms (e.g. paramecium, acellular slime mould, the pea plant *Pisum sativum*, and creeping cinquefoil *Potentilla reptans*) in the absence of neurons or neurocognitive representations in the brain, raising the question of whether, despite differences in evolutionary taxonomy and organisation, there are convergent functional architectures (Brette, 2021; Gruntman *et al*., 2017; Reid *et al*., 2012; Wang *et al*., 2023b).

The organisational properties of decision‐making in many complex systems, including social decision‐making in insects (Dussutour *et al*., 2004) and flocking behaviour of birds (Ballerini *et al*., 2008), apply additional pressure to any principally brain‐based theory of decision‐making, where adaptive behaviour is supported by decentralised or collective regimes that stand in stark contrast to their neural counterparts. Within non‐neural systems like the acellular slime mould *Physarum polycephalum*, alternative biophysical mechanisms exploit cellular structure and dynamics to support adaptive decision‐making, with a central theoretical role proposed for regulatory control, environmental feedback, and dynamic encoding, and with greater emphasis placed on the distributed features of problem‐solving and environmentally embedded factors that constrain decision rules (Baluška & Levin, 2016; Bechtel & Bich, 2021; Bich & Bechtel, 2022; Bleuven & Landry, 2016; Boisseau, Vogel & Dussutour, 2016; Boussard *et al*., 2021; Lyon, 2015; Lyon *et al*., 2021; Van Duijn, 2017).

Early systematic studies already recognised the complexity of unicellular behaviour, with notable continuities between the behaviour of humans and ‘lower’ organisms. Jennings (1906), in a detailed descriptive account of protozoan behaviour, documented consistent patterns of reactivity and movement that were indicative of non‐reflexive capacities, and that dovetailed with a set of ideas and principles we now recognise as some of the mainstream tenets of psychology (see e.g. Bain, 1875; Hobhouse, 1901; Spencer, 1894). This line of research was extended in the late 20th century, when Koshland (1983) characterised bacterial signal transduction as analogically comparable to neuronal processes, thereby connecting microbial responses with frameworks more often applied to animals, whilst Gelber (1952, 1956) undertook meticulous experimentation in order to probe the Pavlovian conditioning responses of trained ciliates. More recently, several researchers have proposed that the empirically grounded behavioural complexity of unicellular organisms may reflect forms of cellular sentience (Baluška & Reber, 2019; Reber, Baluska & Miller, 2023; Reber *et al*., 2024). Overall, the adaptive capacities of both unicellular and multicellular organisms appear to be supported by an extensive range of biophysical mechanisms and organisational control strategies relevant to decision‐making (Armus, Montgomery & Gurney, 2006; Baluška & Levin, 2016; Dussutour, 2021; Eckert *et al*., 2024; Gershman *et al*., 2021; Godfrey‐Smith, 2016; Rajan *et al*., 2023; Reid *et al*., 2012; Saigusa *et al*., 2008; Tagkopoulos, Liu & Tavazoie, 2008; Wang *et al*., 2023b, 2021; Yi *et al*., 2000). The acellular slime mould *P. polycephalum* anticipates periodic events (Saigusa *et al*., 2008) and is able to solve mazes by optimising nutrient acquisition paths in a decentralised manner (Nakagaki, 2001; Nakagaki, Yamada & Tóth, 2000). Other unconventional organisms such as pea plants exhibit risk‐sensitive decision‐making in root growth (Dener, Kacelnik & Shemesh, 2016) and anticipatory, goal‐directed movements in tendril development in response to resource availability (Wang *et al*., 2023b). Even bacteria exhibit anticipatory responses in terms of dynamic adjustments to gene regulation and/or metabolic pathway switching in response to environmental cues [i.e. nutrient availability (Balázsi, Van Oudenaarden & Collins, 2011; Bowsher & Swain, 2014; Perkins & Swain, 2009)].

The above examples suggest that several behaviours may fulfil criteria for decision‐making (e.g. anticipatory and goal‐directed responses to aversive or attractive environmental stimuli), and resonate with the kind of open‐ended, adaptive behaviours, motivations or autotelic purposes that are characteristic of biological organisms more generally. Importantly, many (if not all) of these processes are regulated by molecular and cellular signalling pathways, which pre‐date the evolution of nervous systems, raising the interesting theoretical possibility that decision‐making is an evolutionarily conserved capability, constrained in fundamental ways by biological organisation (Baluška & Levin, 2016; Cisek, 2019, 2022).

To develop an integrative perspective on decision‐making, it is necessary to bridge the conceptual gaps between more traditional cognitive models and ecological and organisational perspectives, and include theoretical and experimental evidence from more unconventional model organisms more explicitly within studies of decision‐making. As Huang *et al*. (2021) argue, expanding the repertoire of model organisms to include phylogenetically diverse systems provides an avenue for understanding the similarities and differences in decision‐making across species, and the salient role of biological organisation in determining how decision‐making is realised under various guises. To this end, we propose the Continual Decision‐Making Dynamics (CDMD) model, which reframes the dynamics of decision‐making in distributed and decentralised systems as a historically embedded regulatory function. Specifically, CDMD builds on organisational theories of biological autonomy (Bich, 2024; Bich & Bechtel, 2022) by formalising how decision strategies emerge from adaptive control mechanisms that do not necessarily require orthodox representational formats. In doing so, we provide a theoretical framework that can be empirically refined so as to understand better alternative modes of decision‐making present across phylogenetic distances.

The CDMD framework offers a general account of decision‐making by treating it as a continuous negotiation of constraints, where each decision reshapes the landscape of future possibilities. This negotiation operates through processes of self‐regulation, constraint propagation, and the embedding of behaviour within variable environmental contexts. In this respect, CDMD resonates with process‐based theories of cognition (Di Paolo, Burhmann & Barandiaran, 2017) and non‐representational approaches to decision‐making (Keijzer, 2017). At the same time, it extends these perspectives by providing a formal model that generates empirical predictions, often absent from more philosophically oriented discussions. The framework thus accounts for decision‐like behaviour as emerging from ongoing, feedback‐driven adaptation, while also supplying a cross‐systems perspective that spans phylogenetically diverse forms of organisation. Finally, by incorporating both state‐dependent (condition‐driven) and process‐dependent (interaction‐driven) factors, CDMD emphasises the organisation of agent–environment dynamics as central to constraining and enabling adaptive strategies.

The paper is structured as follows. We begin by identifying three key theoretical challenges in the attribution of decision‐making to non‐neural organisms: the risk of overgeneralisation, representational assumptions, and ambiguity across levels of organisation (Section II). We then introduce the CDMD framework, and contextualise its formalisation within examples of decision‐making across both neural and non‐neural systems (Section III). Finally, we critically discuss some of the broader implications of CDMD for understanding decision‐making as a widespread property of adaptive systems, as well as current limitations and outstanding challenges for our model (Section IV). Overall, we aim to show how this model has the potential to provide a more integrative theoretical perspective on decision‐making that can account for adaptive strategies in a greater diversity of model systems, and that can be applied to interpret distinct temporal trajectories of organismic functioning.

## II. THREE THEORETICAL CHALLENGES IN STUDYING DECISION‐MAKING IN NON‐NEURAL ORGANISMS

Decision‐making is often treated as synonymous with (neuro)cognitive function in human and non‐human animals (Friston *et al*., 2014; Gallistel & King, 2011; Miller & Cohen, 2001; Shea, Krug & Tobler, 2008; Simon & Newell, 1971). Yet, this is not necessarily the case. In particular, non‐neural organisms exhibit behaviours consistent with the decision‐making label in the absence of some or all of these characteristic features. This raises three fundamental challenges if we want to connect decision‐making with cognitive capacities in non‐neural systems: (*i*) the granularity problem, whereby mechanistically distinct processes are conflated under a single conceptual label, (*ii*) the representation problem, where models typically assume that decision‐making requires internal representations, and (*iii*) the levels of organisation problem, in which adaptive responses occur at multiple biological scales, creating ambiguity when distinguishing decision‐making from more reflexive, low‐level homeostatic or regulatory processes. These challenges motivate a comparative, mechanistically grounded, and testable framework for decision‐making that moves beyond neural assumptions while preserving explanatory precision. This section explores these challenges, and interrogates the conceptual and methodological complexities they introduce when studying decision‐making in non‐neural systems.

### (1) The granularity problem

A central challenge in defining decision‐making lies in its broad and varied application across disciplines. Decision‐making is widely applied across various subfields of neuroscience, biological sciences, ecology, engineering, and philosophy, yet these fields often use the term to refer to radically different constructs and units of analysis (Bechtel & Bich, 2024; Bich & Bechtel, 2022; Levin & Dennett, 2020; Potochnik, 2017). Recall, for example, how cognitive and computational neuroscience model decision‐making as an inferential or evidence‐accumulation process (Collins & Shenhav, 2022; Gold & Shadlen, 2007), whilst behavioural ecologists describe it as involving state‐dependent optimisation related to fitness trade‐offs (Houston & McNamara, 1999; Stephens & Krebs, 1987). Furthermore, decision‐making processes can be implemented in a seemingly infinite number of ways through algorithmic models, not only in biological neural networks but also in artificial systems and contexts, *via* Markov decision processes or reinforcement learning (RL) architectures (Bellman, 1957; Niv, 2009; Sutton & Barto, 1998), which we often deliberately use to theorise and model our own behaviour in a faithful (although approximate) manner. Perhaps most troublingly, non‐neural organisms like slime mould, bacteria, and plants exhibit analogous decision‐making behaviours with vastly different substrates, reviving a host of philosophical problems and quandaries related to substrate dependence and multiple realisability of cognitive functions (Dussutour *et al*., 2010; Gruntman *et al*., 2017; Reid *et al*., 2016).

These differences introduce a tension when attempting to define decision‐making in any generalisable yet meaningful sense. If defined too liberally, and in the absence of empirical validation, decision‐making risks encompassing nearly any form of adaptive response and conflating more relevant (or genuine) adaptive behaviour with simple stimulus–response actions and reflexes, diluting its explanatory power and leading us perhaps to discredit more plausible accounts or demonstrations. On the other hand, if defined too restrictively, certain biases (e.g. selection biases) are introduced and reinforced within empirical practices, and we may end up overlooking potential sources of insight into decision‐making; this can be thought of as a kind of epistemic blind‐spot, wherein our knowledge and understanding of cognitive phenomena like decision‐making is obscured by the paradigmatic human case.

Instead, we propose a middle path, by which decision‐making is an organisationally grounded, mechanistically specified set of processes that depend on the self‐regulatory, history‐dependent dynamics of a system, yet does not deny the relevance of cognitive or computational mechanisms that typically support motifs of decision‐making. Importantly, this approach pushes towards epistemic flexibility and pluralism with respect to the utility of available models, where different model organisms likely require different decision‐making models, and multiple theoretical lenses are necessary to understand its strategic diversity across disparate phyla. Furthermore, we also propose empirical tests and falsifiable predictions of the CDMD, particularly in cases where predicted outcomes may deviate from alternative models of decision‐making (see Section IV). This allows us to arbitrate amongst the models that most plausibly apply to target phenomena.

### (2) The representation problem

A second challenge lies in the association between decision‐making and representational models of cognition. Human‐centric cognitive models frequently assume that solving problems through decision‐making requires a system to consult and operate over an internal model of the world (e.g. a kind of mental map), which allows an agent to select actions based on predictions about its future state given the spatial or temporal characteristics of the environmental niche in which such actions are situated, whether through planning or simulation‐based procedures (Friston *et al*., 2018; Gallistel, 2001; Van Der Meer, Kurth‐Nelson & Redish, 2012). Such internal representations are typically considered as essential theoretical posits for explaining cognitive behaviour within ‘representation‐hungry’ domains like planning and decision‐making (Kiverstein & Rietveld, 2018). In human and (some) non‐human animals, these behaviours are assumed to be structured through hierarchical models of executive function, whereby decision strategies are regulated from the top down, allowing for flexible control over actions, and the development of domain‐specific knowledge and skill acquisition. However, not all adaptive choices require planning over such internal models of the environment, relying instead upon alternative strategies to resolve exploration–exploitation trade‐offs, whether exploiting the learned value of previously chosen actions to enable efficient decision‐making on a known task, strategic variability of behaviour, or some arbitration between model‐free and model‐based modes of control on the basis of the contingent demands associated with a given environment or task (Held *et al*., 2025; Reynders, Verguts & Braem, 2025). Furthermore, non‐neural systems appear to rely on distributed mechanisms and feedback control rather than internal models or top‐down structure, exploiting environmental structure through biophysical processes and proto‐cognitive mechanisms to enable adaptive decision‐like behaviour (Barandiaran & Moreno, 2006; Van Duijn, Keijzer & Franken, 2006).

Anticipation, for instance, is often associated with sophisticated abilities to simulate future states internally and plan accordingly (Friston *et al*., 2018). Yet, the presence of anticipatory behaviour in non‐neural systems challenges this assumption. In the case of the acellular slime mould *P. polycephalum*, anticipatory responses are thought to be exhibited in adjustments to foraging patterns based on prior experience, depositing chemical gradients that allow it to map out the recurrence of environmental cues (Nakagaki *et al*., 2000; Saigusa *et al*., 2008). *P. polycephalum* appears to accomplish these feats through the physical propagation of oscillatory signals and offloading of extracellular slime trails, rather than deliberative reasoning, allowing for the externalisation of path structure and decision history. Additionally, pea plant exhibits a form of anticipatory, decision‐like behaviour through goal‐directed circumnutations in its tendrils, and appear to exhibit preference‐sensitive choice behaviour (Guerra *et al*., 2019; Wang *et al*., 2023a, 2021).

Such cases challenge representationalist assumptions (and those that concern centralised control of adaptive behaviour), suggesting that anticipation also emerges as a functional property of distributed or decentralised systems (Keijzer, 2003; Lyon *et al*., 2021; Van Duijn *et al*., 2006; Wang *et al*., 2021). Just like the behaviour of humans and other animals, these apparent expressions of decision‐making are thought to depend on regulatory control mechanisms, which include but are not limited to self‐organised, distributed feedback (Bich, 2024). More generally, understanding how control architectures are (approximately) organised across systems [e.g. within hierarchical (Swanson, 2000) or heterarchical (Bechtel & Bich, 2021; Huebner & Schulkin, 2022) regimes] helps to identify salient differences in the mechanisms that support adaptive behaviour, and to model the purported variation of cognitive function across the tree of life. For example, in hierarchical architectures, regulation is structured in layered levels, where higher levels direct or constrain the operations of lower ones in a relatively stable top‐down manner. By contrast, heterarchical architectures distribute control across interacting components, such that regulation arises from reciprocal and context‐dependent interactions without a fixed chain of command. We propose that these distinct forms of organisation constrain decision rules and strategies in different ways, shaping how trade‐offs are resolved across levels of organisation and complexity, particularly as a function of task environment and feedback control.

### (3) Ambiguity in levels of organisation

A third (and related) challenge concerns how decision‐making operates across multiple biological scales both within and across individuals. Here, ambiguity is introduced through efforts to distinguish between ‘higher‐level’ cognitive decision‐making and ‘lower‐level’ or reflexive regulatory responses (Bechtel & Bich, 2021; Levin, 2019). Adaptive responses at cellular, tissue, and organismal levels of analysis illustrate this challenge. Decision‐making in neural systems is thought to involve cognitive mechanisms for evidence accumulation and reinforcement‐guided action selection, with considerable evidence of brain‐level mediation by neocortical regions such as the prefrontal cortex and more phylogenetically ancient structures like the basal ganglia (Schultz *et al*., 1997; Shenhav *et al*., 2013). At the microcellular scale of the organism or plant, the situation is quite different. Here, local structures in climbing plants, for example, also integrate diverse environmental feedback signals (e.g. light, nutrients), adjusting global shoot/root growth patterns and morphological structure in a decentralised manner in order to maintain physiological integrity. Distinguishing between such cases of decision‐making in terms of regulatory processes thus requires a conceptual framework sensitive to the organisational autonomy of different biological scales. More generally, a regulatory approach to cognition suggests that decision‐making should be understood in terms of autonomy‐preserving mechanisms that allow organisms to select actions based on their needs and environmental constraints (Bich, 2024).

Like Huebner & Schulkin (2022), we suggest that one productive distinction that can contextualise these different control strategies is to characterise how, when individual cells within multicellular organisms respond adaptively, they nonetheless operate within systemic constraints, and lack the autonomous decision‐making characteristics of unicellular or whole‐organism contexts. We suggest that such distinctions help to clarify decision‐making as an organisationally grounded phenomenon, and how regulatory thresholds shift the system from one stable state to another in a context‐sensitive manner (Bich *et al*., 2016).

## III. A THEORETICAL APPROACH TO CONTINUAL DECISION‐MAKING DYNAMICS

The CDMD addresses the aforementioned challenges by aiming to account for three major characteristics of decision‐making: (*i*) its grounding in biological self‐regulation, where organisms integrate past states, present constraints, and anticipated futures (*organisational structure*); (*ii*) its recursive, history‐sensitive construction, where prior decisions shape subsequent decision possibilities through path‐dependent modifications (*temporal structure*); and (*iii*) its regulatory functioning, which we propose enables transitions between stable states. These transitions occur through ongoing adaptive interactions with the environment, rather than through fixed representational computations (*regulatory transition*). Together, these characteristics provide a more nuanced view of decision‐making across both neural and non‐neural systems as an ongoing negotiation between internal organisational constraints and external environmental fluctuations. Furthermore, we aim to show how these characteristics enable the formalisation of decision‐making across a greater range of adaptive systems whilst preserving the explanatory potency of the framework.

To substantiate this perspective, we introduce a formal model that explicates the recursive structure of decision‐making within the CDMD framework. Specifically, decision‐making is defined as a temporally extended, recursive process where the state of the system at a given moment is determined by prior decisions, historical constraints, and environmental conditions. Let: $S_{t}$ represent the system state at time t, a dynamic configuration shaped by prior regulatory modifications including both internal and external conditions; $E_{t}$ represent the environmental conditions at time t; $O_{t}$ be the outcome resulting from action $A_{t}$ in state $S_{t}$, where $A_{t}$ refers to the action or decision performed at time t; and $T_{t}$ represent the *time‐print*, a historically embedded constraint defined as the cumulative trace of past outcomes. $T_{t}$ records how prior decisions constrain and shape future trajectories (further details of its formal role are developed in Section III.2).

The recursive state update function, defining how decisions unfold dynamically, is given by:

(1) $$S_{t+1}=f\left(S_{t},O_{t},E_{t+1},T_{t}\right),$$

where f represents the way prior states ($S_{t}$), outcomes $O_{t}$, current environmental conditions $E_{t+1}$, and historical trajectories ($T_{t}$) collectively shape the next system state. This recursive structure ensures that decision‐making is path dependent and continuously reshapes the adaptive landscape. In the next subsections, we discuss the key features of the CDMD framework, their formalisation, and how the framework is integrated into example studies.

### (1) Organisational grounding of decision‐making

The first major characteristic of decision‐making according to the CDMD framework is its *organisational structure*. Here, we build on organisational theories of biological autonomy (Bich & Bechtel, 2022; Moreno & Mossio, 2015) to decision‐making as a structurally constrained yet dynamically evolving process. Unlike computational models that treat decision‐making as an abstract problem of inference, the CDMD views decision‐making as a function of regulatory architectures that maintain system integrity across time (Bich, 2024). According to these accounts, decision‐making emerges from an organism's (or system's) ability to regulate its own interactions with the environment, ensuring the balance between structural integrity and adaptive flexibility (i.e. self‐regulation). These regulatory properties of decision‐making thus allow us to view decision trajectories as involving a kind of interactive reciprocity that holds between system states, dynamics over processes, and behavioural outcomes, such that modifications to the system are produced on the basis of feedback. Thus, for each decision event, the system's adaptive landscape is effectively altered, ensuring that future decisions are contextually situated and historically non‐reversible. In this context, non‐reversibility does not imply that outcomes cannot be undone or that a system cannot later ‘reverse’ its behaviour in accordance with goals (as in typical instances of feedback control in self‐organising systems). Rather, it indicates that each decision leaves a time‐print ($T_{t}$) that irreversibly becomes part of the system's trajectory: even when subsequent behaviour compensates for or counteracts a prior outcome, the reversal itself constitutes a new imprint, thereby embedding decision‐making within a history‐sensitive process. Whilst state‐based cognitive models (e.g. where beliefs and utility functions determine optimal choices) can be positioned as particular instantiations of the decision‐making process, we consider the CDMD as able to account better for the primitive roots of decision‐making in more holistic biological processes.

To capture this formally, we define decision‐making as a regulatory transition between system states. The system state $S_{t}$ is not merely a snapshot of internal or external conditions, but a dynamic configuration shaped by prior regulatory modifications. The action/decision ($A_{t}$) selection process can thus be represented by the function:

(2) $$A_{t}=g\left(S_{t},E_{t},T_{t}\right),$$

where g describes how the system selects an action based on its current state, environmental context, and historical imprints encoded in time‐prints. The outcome $O_{t}$ of a given action is then defined as:

(3) $$O_{t}=h\left(A_{t},E_{t},T_{t}\right),$$

where *h* is a causal–stochastic mapping that determines the regulatory outcome generated by the system's action in its current state and environmental context. This outcome then generates a new time‐print:

(4) $$T_{t+1}=\Phi \left(T_{t},O_{t}\right),$$

where $\Phi \left(T_{t},O_{t}\right)$ represents a memory encoding function, modifying the historical trajectory based on new outcomes, determining how strongly past outcomes influence current decisions. In experimental scenarios, Φ could involve exponential decay functions, reflecting how biochemical markers or environmental traces fade over time, or relate to other parameters such as weighting of recent *versus* distant events, or signalling thresholds. This recursive formulation characterises each decision as both informing and constrained by prior decisions, embedding path dependence into the system.

Although the decentralised assumptions of the CDMD explicitly depart from representational stances typical of cognitive models, it remains amenable to more gradualistic forms of minimal or structural representations, whereby representational content emerges from system–environment interactions and structural correspondences rather than explicit symbolic structures. In principle, CDMD's historically embedded constraints (‘time‐prints’) could be interpreted as minimally representational, encoding functional or structural mappings implicitly through distributed biochemical or environmental processes. However, CDMD neither relies explicitly on this representational interpretation nor requires minimal representation to explain adaptive behaviour. Instead, our formalism highlights historically recursive constraints explicitly without commitment to representational interpretations. Future empirical work could explicitly test whether adaptive dynamics observed within CDMD contexts can be fully captured by non‐representational dynamics or whether minimal representational interpretations offer additional explanatory or predictive value.

### (2) Time‐prints and embedded constraints

Building on the organisational structure of decision‐making, the second major characteristic according to the CDMD framework is its *temporal structure*. Crucially, decision‐making in biological systems is both a process and an outcome, shaped by an organism's history, anticipatory capacities, and environmental interactions. As a process, decision‐making unfolds as a temporally extended integration of sensory inputs, past states, and regulatory feedback. This process operates across multiple nested timescales, where rapid feedback loops interact with slower, cumulative adaptations (Bich, 2024). Conversely, as an outcome, each decision event reconfigures the system's future state‐space, shaping how subsequent decisions unfold. To formalise this, we introduce *time‐prints*: historically embedded constraints that regulate future decision pathways without requiring explicit memory representations. Each time‐print $T_{t}$ is defined as a cumulative function of past outcomes, where the outcome of one decision simultaneously serves as the process input for the next sequence as mentioned in Section III.1:

(5) $$T_{t+1}=\Phi \left(T_{t},O_{t}\right),$$

where Φ represents a historical transformation function encoding prior decision constraints. Unlike symbolic memory, time‐prints are distributed across biochemical, structural, or environmental modifications, ensuring that decision‐making remains contextually embedded. The recursive process‐outcome function defining decision trajectories thus extends as:

(6) $$S_{t+1}=f\left(S_{t},O_{t},E_{t+1},T_{t}\right),$$

which ensures that each decision modifies the system's future state space, reinforcing historical imprints. Importantly, the function Φ could incorporate temporal decay rates α, reinforcement factors β, and weight distributions, reflecting how prior influences persist or fade over time:

(7) $$T_{t+1}=\alpha T_{t}+\beta O_{t},$$

where 0 < *α* < 1 governs memory persistence, and *β* represents the weight of recent *versus* distant events. A time‐print here is thus construed as a process–outcome pair that encodes the constraints imposed by past decisions, shaping the system's future decision trajectories. Unlike fixed timestamps, these time‐prints aim to contextualise decision‐making within a system's ongoing adaptive history, ensuring that each decision is situated within a broader trajectory of organism–environment interactions. To demonstrate how time‐prints could work in principle, we can consider some empirical examples of their operation within biological systems, particularly in biological systems where decision‐making appears to emerge through non‐representational, process‐dependent mechanisms.

**Fig. 1:**
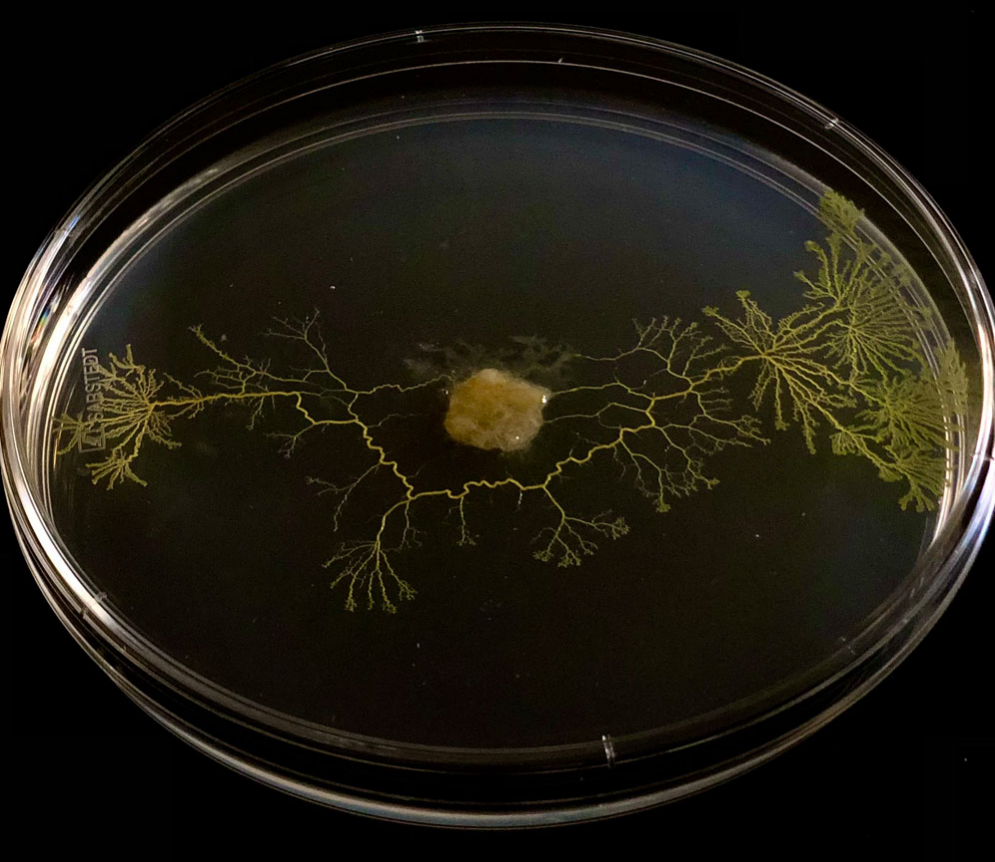
The acellular slime mould *Physarum polycephalum* effectively embodies the concept of time‐prints through decision‐making behaviour. The deposit acts as a history‐dependent constraint, with tangible effects on where the organism will forage in the next action sequence, with successive decisions forming a temporal chain of time‐prints.

One of the most apt depictions of historical imprints relevant to non‐neural systems is the optimal foraging strategy of the acellular slime mould (Fig. 1). *P. polycephalum* explores its environment (E) by expanding and retracting search fronts (A) in an oscillatory manner, with more efficient tubules of its biomass forming a ‘chemical map’ that reflects the system's current state (S) and resembles precursors of internal memory networks (Chung & Choe, 2009; Miyake *et al*., 1994). These organisms have been shown to solve spatial navigation problems by depositing extracellular slime trails that encode the outcomes (O) of prior exploratory actions, particularly with respect to nutrient quality (Nakagaki *et al*., 2000; Reid *et al*., 2012). The slime trail both records past states and actively influences future choices by repelling the organism from inefficient (or suboptimal) paths already taken. Building on this work, we propose that these extracellular trails function as environmentally embedded time‐prints (T), regulating future action, in the absence of any internal memory storage. Here, memory is distributed and non‐symbolic, encoding outcome variables related to decision history through system‐wide state transitions and environmental imprints, which can be viewed as analogous to (although distinct from) neural memory and synaptic modifications (Bonabeau, Dorigo & Theraulaz, 1999; Boussard *et al*., 2021; Kramar & Alim, 2021; Pfeifer & Bongard, 2006). These distributed regulatory structures exhibited during foraging decisions allow for the adaptive resolution of trade‐offs given certain constraints in the absence of centralised control or, indeed, encephalisation, including trade‐offs over response times and accuracy (e.g. Latty & Beekman, 2011).

Past movement can therefore alter future choices, creating a history‐sensitive decision architecture that depends not on symbolic processing or centralised control, but on heterarchical forms of control, i.e. distributed regulatory architectures in which influence emerges from reciprocal interactions among components and their environment. In this respect, the CDMD proposes that memory structures can be distributed, processual, and recursively embedded within environmental interactions, consistent with previous work demonstrating the dynamic encoding of information through intracellular oscillators (Fleig *et al*., 2022; Takamatsu, Fujii & Endo, 2000; Takamatsu, Takaba & Takizawa, 2009; Tsuda & Jones, 2011).

### (3) Regulatory function across levels of organisation

The CDMD framework also resolves ambiguities related to decision‐making across different biological scales by defining decisions as *regulatory transitions* between stable system states. This approach clarifies how organisational dynamics at cellular, organismal, and collective levels support decision‐making, despite distinct underlying mechanisms and functional plasticity. Indeed, one core strength of the CDMD and its concept of time‐prints is its ability to capture the adaptive decision‐making dynamics of both individuals and collectives. In this context, we formalise path dependence, ensuring that two systems with identical present states but different histories will diverge in their decision‐making. This was previously captured mathematically in Equation (2) as: $A_{t}=g\left(S_{t},E_{t},T_{t}\right)$ where the selection of an action $A_{t}$ depends not only on the present state $S_{t}$ and environment $E_{t}$, but, crucially, on the accumulated historical imprints $T_{t}$. Given this dependency, we define a path‐dependent decision function:

(8) $$A_{t}=g\left(S_{t},E_{t},T_{t}\right)\neq g\left(S_{t},E_{t},T_{t-1}\right),$$

which formalises how past decisions modify future options. The degree to which history influences decision‐making can be quantified *via* a weighting parameter *γ*, which modulates sensitivity to past states:

(9) $$P\left(A_{t}|S_{t},E_{t},T_{t}\right)=\frac{e^{\mathrm{\gamma U}\left(A_{t}|S_{t},E_{t},T_{t}\right)}}{\sum_{A^{\prime }}e^{\mathrm{\gamma U}\left(A^{\prime }|S_{t},E_{t},T_{t}\right)}},$$

where $U\left(A_{t}\right)$ represents the utility of an action conditioned on historical imprints, and.

$A^{\prime }$ denotes the set of all feasible actions available to the system at time t. When γ → 0, past decisions exert minimal influence, whereas higher γ values encode stronger historical constraints. This recursive formulation enables decision pathways to emerge dynamically rather than being predetermined, ensuring that decision histories shape future action landscapes in a manner that integrates past regulatory structures.

A beguiling example of this can be observed through starling (*Sturnus vulgaris*) murmurations: fluid aerial formations created by thousands of individual starlings in coordinated flight. In the final moments of sunlight in the autumnal skies over Brighton seafront, spectators of these beautiful displays have pondered how and why thousands of these birds perform their elaborate twists and turns in collective unison, with flocks continuously changing their shape, size, and internal structure over the ruins of the West pier. We can contextualise this spectacle of emergent behaviour, believed to result from predation behaviour, in terms of continual decision‐making dynamics, and in particular, by appealing to our notion of time‐prints (T).

Murmurations involve rapid coordination without centralised control, emerging through localised interactions wherein each bird dynamically adjusts its velocity and direction (A) based on its neighbours' movements (Ballerini *et al*., 2008; Cavagna *et al*., 2013). The collective behaviour (O; i.e. ‘flocking’) of European starlings has been well documented, and appealed to as an example par excellence for understanding the emergent dynamics of complex systems (Parisi, 2023; Vallee, 2021). Here, each individual will align its flight direction with that of its neighbours (usually the six or seven nearest neighbours), a rule that gives rise to collective motion within the group, and continuous changes in the neighbouring partner, propagating information transfer across the flock in a rapid manner. In this context, time‐prints refer to the historical imprints of individual actions (A) within the flock, with the previous state (S) of the flock (hysteresis) influencing and shaping collective decisions. For example, if a segment of the flock veers in one direction, this change propagates through the group, altering the flock's structure and informing subsequent movements in a processual, history‐dependent way.

This local interaction model allows the flock to respond rapidly to environmental cues (e.g. predation) without centralised control, with emergent patterns that are not pre‐planned, but instead formed dynamically in accordance with each bird's position and velocity in real time. These interactions create a form of collective memory analogous to time‐prints, where the flock learns how past configurations of the system influence current and future movements. When a predator approaches, the initial evasive manoeuvres by individual starlings create perturbations that propagate through the flock, thus serving as temporal markers of past events that inform the flock's ongoing dynamics and allowing for adaptive responses without disrupting overall integrity of the collective unit, whilst allowing the collective to return to a more stable state. These adaptive, decentralised motions are thought to ensure cohesion and survival, such that historical interactions (time‐prints) are embedded in the flock's ongoing dynamics rather than a visible trace of the flock's decision history.

### (4) Integrating state‐dependent and process‐dependent dynamics

The view put forward within the CDMD framework captures continuous realignment of decision pathways. The decision strategies at work here are not only shaped by internal states (e.g. metabolic demands, sensory processing), but also by external environmental fluctuations that require real‐time calibration or adjustments (i.e. decisions are contextually situated). To formalise this, we introduce an adaptive landscape function $L_{t}$ that evolves recursively based on past outcomes:

(10) $$L_{t+1}=\delta L_{t}+\epsilon O_{t},$$

where *δ* determines the persistence of prior structural conditions and ϵ represents the extent to which new outcomes reshape the adaptive space. This function encodes how prior interactions modify future possibilities, ensuring that decision trajectories remain embedded within an evolving regulatory framework. The CDMD framework thus enables a more generalised approach to understanding decision‐making across biological systems by integrating state‐dependent (condition‐based, captured by system states $S_{t}$ and environmental conditions $E_{t}$) and process‐dependent (interaction‐based, captured by time‐prints $T_{t}$ and outcomes $O_{t}$) factors. The CDMD also explicitly highlights how each outcome modifies future decision‐making trajectories through a recursive state‐update function:

(11) $$S_{t}=f\left(S_{t-1},O_{t-1},E_{t},T_{t-1}\right),$$

where outcomes $O_{t-1}$ feed back into the next state $S_{t}$ *via* historical constraints $T_{t-1}$. Each action thus alters the adaptive landscape for subsequent decisions. Practically, this means historical interactions are encoded as changes in the physical environment (e.g. chemical gradients, extracellular slime trails, or collective pattern formation) or internal state dynamics (e.g. biochemical or mechanical configurations), which directly modify the future decision space. In other words, previous adaptive interactions reshape future possibilities because each action–outcome pair leaves an ‘imprint’ that changes how subsequent states and actions are evaluated. For example, when slime mould explores a nutrient‐rich path, the slime trail it leaves behind biases future movements away from previously exploited paths. To formalise this explicitly, we define a state‐space transformation explicitly linked to historical interactions. Specifically, each historical outcome reshapes a dynamic, adaptive landscape function $L_{t}$:

(12) $$L_{t}\left(S_{t+1},A_{t}\right)=f_{L}\left(L_{t-1}\left(S_{t},A_{t-1}\right),O_{t}\right),$$

where $L_{t}\left(S,A\right)$ is an adaptive landscape function describing the feasibility, costs, or benefits of state‐action pairs at time *t*; $f_{L}$ is a transformation function updating the adaptive landscape based explicitly on the previous landscape $L_{t-1}$ and the current outcome $O_{t}$; and $O_{t}$ encodes structural/environmental changes (e.g. biochemical deposition or spatial modification) from actions at time *t*, altering accessibility or attractiveness of future states and actions. Importantly, while $E_{t}$ refers to the immediate environmental parameters influencing outcomes at each time step, $L_{t}$ captures how these outcomes recursively integrate such influences into a history‐dependent structure that constrains future decision possibilities.

For computational tractability, assume $f_{L}$ is a linear or non‐linear update rule, e.g.:

(13) $$L_{t}\left(S_{t+1},A_{t}\right)=\delta L_{t-1}\left(S_{t},A_{t-1}\right)+\mathrm{\epsilon M}\left(O_{t}\right).$$

Parameter *δ* (with 0 < *δ* ≤ 1) modulates the persistence of past landscape structure, whilst parameter ϵ determines the strength of new structural or environmental modifications $M\left(O_{t}\right)$. This adaptive landscape explicitly encodes the idea that each decision dynamically reshapes future possibilities, capturing the formal notion of historical interactions. Future possibilities thus emerge explicitly as reshaped by historical interactions through these recursively evolving adaptive landscapes.

### (5) Empirical predictions of CDMD

The CDMD framework provides clearly specified empirical predictions, which can be explicitly tested across diverse biological model systems. Here, we briefly articulate these predictions and propose practical experimental designs to test CDMD directly. Specifically, CDMD makes three empirically testable predictions:

*Prediction 1*: historical constraints. Altering or disrupting historical traces (time‐prints; e.g. extracellular trails in slime moulds) should result in measurable changes in future adaptive decisions compared to intact control conditions. For example, experimentally erasing slime mould trails at distinct time intervals would be predicted to shift the subsequent foraging decisions and spatial distribution of biomass explicitly away from established paths.
*Prediction 2*: environmental encoding. Removing or significantly modifying environmental structures (e.g. extracellular chemical gradients or environmental markers) should systematically alter an organism's adaptive trajectory. In practical terms, root growth experiments could explicitly disrupt previously explored soil patches (removal or chemical masking of root exudates), observing measurable differences in adaptive root exploration patterns, speed, and efficiency compared explicitly to controls.
*Prediction 3*: conflict resolution of historical *versus* immediate rewards. When historical constraints explicitly conflict with immediate environmental rewards (such as previously established trails *versus* newly introduced nutrient gradients), CDMD explicitly predicts measurable historical biases. This prediction could be empirically tested by offering slime moulds established but degrading nutrient trails *versus* novel nutrient‐rich sources. Observing whether the organisms initially persist in following historical imprints, despite immediate environmental incentives, would explicitly test CDMD's historical‐dependency predictions. Furthermore, collective animal systems such as starling murmurations also offer practical, empirical settings to test CDMD's predictions explicitly. Naturalistic perturbation experiments, such as tracking historical flock trajectories before and after explicit disturbances (predator introduction or auditory perturbation), would allow researchers to test whether flock adaptive decisions display measurable historical persistence, as CDMD predicts.

## IV. DISCUSSION

The CDMD framework aims broadly to account for the distributed processing of decisions across biochemical, biophysical, and environmental modifications of biological systems, and thus, the function of decision‐making as an embedded regulatory structure. As such, decision‐making remains both temporally continuous and path dependent. Importantly, such a characterisation allows for comparative insights across neural and non‐neural organisms, applying to diverse examples from unicellular organisms to multicellular collectives (i.e. is applicable across organisations). Here, decision history is not stored as an abstract representation but as an active component of future decision‐making, shaping the organism's behavioural trajectory in path‐dependent ways. By relying on distributed, non‐symbolic memory structures (time‐prints), the model naturally captures decision‐making strategies that emerge from embedded interactions rather than centralised cognitive architectures or explicit internal representations. Indeed, the CDMD possesses a distinctive ability to characterise the adaptive behaviours exhibited by non‐neural organisms and collective animal behaviour, such as starling flocks, all of which employ decentralised, environmentally embedded forms of regulation.

Importantly, the CDMD is sympathetic to considerations of ecological psychology and dynamical systems theory, which have addressed adaptive behaviour without strict representational assumptions, placing greater emphasis on continuous organism–environment interactions that depend on non‐representational, self‐organising adaptive dynamics (Beer, 2021; Chemero, 2013; Di Paolo *et al*., 2017; Gibson, 2014). However, the CDMD framework diverges from these research traditions in several important respects. In particular, the CDMD explicitly formalises how past organism–environment interactions constrain future adaptive possibilities, integrating historical interactions as constraints over decision‐making dynamics and embedded within recursive adaptive processes. Furthermore, while our CDMD framework explicitly avoids traditional symbolic representations, it does not necessarily preclude minimal or emergent forms of representational processes (e.g. minimal, structural, or action‐based representations), provided these are understood strictly in terms of functional mappings or historically embedded interactions (Godfrey‐Smith, 2016; Keijzer, 2017; Shea, 2018).

Relatedly, it is important not to over‐generalise the CDMD. One concern is that examples of decision‐making according to the CDMD may in certain cases collapse into more generic descriptions of adaptive regulation. To prevent this, we introduce three explicit mechanistic constraints that define decision‐making under the CDMD framework. First, decisions must be shaped by non‐reversible historical constraints that accumulate over time, altering future state‐space configurations. Unlike simple reflexes or stimulus–response mappings, CDMD requires that past interactions dynamically modify future decision landscapes. Clarifying the robustness, persistence, and functional relevance of distributed memory structures will require precise experimental criteria and measurable proxies, such as biochemical gradients, persistent environmental modifications, or structured behavioural interactions. Second, decision processes must exhibit flexibility in action selection, rather than following rigid stimulus–response mappings to ensure that decision trajectories are not pre‐programmed or fully deterministic. Finally, decision‐making must involve history‐sensitive regulatory modifications that persist over extended timescales, influencing future adaptation to ensure that CDMD applies only to cases where adaptive processes reshape future action spaces in a structured yet flexible manner. This sets it apart from stimulus–response behaviours, homeostatic adaptation, or simple regulatory control mechanisms.

Additionally, systems suited to CDMD exhibit historically sensitive adaptation, distributed or decentralised control architectures, and flexible rather than rigid stimulus–response behaviour. Nevertheless, certain boundary cases plausibly highlight the constraints of CDMD's applicability. First, highly centralised neural decision‐making may indeed exceed CDMD's explanatory strengths, as centralised hierarchical control and explicit representation fall outside CDMD's distributed assumptions. Second, purely reflexive, rigid stimulus–response systems (e.g. fixed action patterns in certain insects or stereotyped reflexes in animals) do not meet CDMD's criteria for adaptive flexibility or historical constraint sensitivity. Third, CDMD depends explicitly on durable environmental embedding or time‐prints, such that any system lacking environmental persistence or exhibiting rapid decay of historical traces may render empirical detection of CDMD dynamics infeasible. Fourth, CDMD places its emphasis on external, history‐dependent decision constraints, and does not, in its present form, adequately capture an organism's ability to interpret, evaluate, and prioritise different decision trajectories based on internal goals, expectations, or preferences. In other words, CDMD currently externalises decision‐making by treating it as an emergent regulatory process rather than a process mediated by subjective evaluation. This is particularly problematic for systems where internal preferences modulate behavioural choices in ways that cannot be explained purely by environmental or historical constraints.

Addressing these limitations proactively, we suggest potential extensions of the CDMD that can complement hierarchical representational models, serving as a hybrid explanation integrating recursive historical constraints alongside centralised control dynamics. Additionally, explicit empirical criteria (e.g. manipulating behaviour *via* historical constraints) could allow researchers rigorously to test CDMD's applicability or identify contexts requiring alternative explanations. Future theoretical developments should further specify substrate‐specific constraints, refining CDMD's explanatory scope and practical utility.

A further, more general conceptual and methodological limitation of the present framework concerns the role of model organisms in studying decision‐making. Importantly, as noted by Levy & Currie (2015), model organisms should be distinguished from theoretical models. Whilst both are called ‘models’, the epistemic mechanisms through which model organisms and theoretical models generate knowledge are different. Theoretical modellers deliberately design and control features in an attempt to mirror a target system or phenomenon, while model organisms are real biological entities, standardised and adapted for research, although notably existing on their own (often unpredictable) terms. This asymmetry between the nature of models as designed through theoretical practice *versus* biological entities as empirical objects of inquiry resists attempts to unify models of decision‐making under a single theoretical framework, and instead supports the use of discipline‐specific epistemologies.

For example, within the CDMD, decision‐making is made explicit at a processual level, clarifying which aspects of a model organism's behaviour support or undermine generalisation of cognitive explanations. In terms of its formalisation, it highlights transferable features or properties of decision‐making or their negation in different systems (e.g. ‘recursion’, ‘time‐prints’), allowing empirical findings in specific species to be situated within a process‐based formalism rather than presumed to extrapolate wholesale, refining the minimal form of the CDMD through a process of ‘weighted feature matching’ (Weisberg, 2012) to distinguish features relevant for generalisation from those requiring recalibration. Indeed, other work has considered the value of such an approach to decision‐making, showing that expanding the phylogenetic scope of model organisms can facilitate the identification of decision mechanisms often masked in human‐centric studies (Huang *et al*., 2021). The formal characteristic of CDMD provides a concrete scaffold for comparison and integration of decision mechanisms, while attempting to maintain sensitivity to each organism's organisational constraints and species‐specific differences. Nevertheless, whilst model organisms serve as empirical entry points into the core organisational features of decision‐making, it is worth re‐emphasising that model organisms are not themselves theoretical models (Levy & Currie, 2015; *cf*. Weisberg, 2012).

A core requirement for any decision model is the ability to make distinct empirical predictions that could be falsified through empirical tests. To bridge this gap, one resolution would be to take an experimental approach that interrogates how organisms internally evaluate trade‐offs, resolve conflicts between historical imprints and immediate needs, and adapt flexibly to prediction errors. Within the CDMD, we can suggest at least three plausible hypotheses that in principle can be falsified (or supported) by empirical evidence. Namely, given the hypothesised CDMD path‐dependent history effects, prior actions will persistently alter future decisions, even when immediate incentives change.

Here, if we perturb past choices, we can test if organisms continue to follow historical imprints despite a changed environment. In slime moulds, a scenario in which established nutrient trails are systematically degraded or overridden by new cues could reveal the extent to which internal states modulate historical biases. Second, the environmental encoding of past actions, whereby history is embedded in structural modifications, will affect future decision‐space. To test this, we can remove or disrupt environmental cues (e.g. erase slime mould trails, alter root exudates) and observe if behaviour still follows past trajectories. Finally, if decisions emerge from recursive constraints rather than explicit evaluation, then by introducing conflicting incentives, history‐dependent constraints should override immediate reward‐based decisions. Eliciting trade‐offs could also provide insight into how internal priorities interact with historical dependencies, where, for example, the speed and structure of an organism's adaptive responses would indicate whether internal priorities (e.g. nutrient stress levels) modulate sensitivity to past imprints. More generally, by providing clearly defined quantitative measures and operational criteria for key constructs, the CDMD provides specific metrics such as temporal decay rates of biochemical markers, statistical dependencies between past and future states, and thresholds for adaptive state transitions. By clearly specifying these metrics, the framework enhances its prospects for testability and precision, and provides explicit pathways for hypothesis‐driven research.

Finally, by incorporating these experimental approaches, CDMD can be extended beyond an externalist regulatory framework, allowing us to examine empirically how organisms actively negotiate competing constraints, resolve uncertainties, and prioritise decision trajectories in order to reveal underlying evaluative and interpretive processes. This will be essential for developing a more comprehensive, integrative account of decision‐making – one that preserves the strengths of CDMD's history‐sensitive, recursive structure while also capturing the rich internal dynamics that shape decision outcomes. To address these potential extensions, future developments of the framework could incorporate explicit internal valuation parameters or motivational gradients (i.e. internal factors) that would explicitly bias adaptive trajectories, including in terms of physiological conditions such as energetic needs, metabolic state, or reproductive motivations. For example, internal valuation parameters could explicitly modulate the strength, persistence, or decay rates of historical constraints (‘time‐prints’) based upon the organism's internal conditions. Empirically, this explicit integration would allow researchers to test how adaptive decisions change in response to internal manipulations (e.g. nutritional or energetic status changes in slime mould). Such an explicit extension of CDMD could bridge environmental and ecological context dependency with intrinsic motivational states, offering richer predictive and explanatory power across diverse biological systems.

## V. CONCLUSIONS

(1) We addressed three key theoretical challenges in studying decision‐making in non‐neural organisms: the granularity problem, the representation problem, and ambiguity in levels of organisation.
(2) The Continual Decision‐Making Dynamics (CDMD) framework offers a formal and integrative model of decision‐making as a historically embedded, organisationally grounded, and temporally extended process.
(3) CDMD demonstrates that both neural and non‐neural systems can support decision‐making through distributed, decentralised, and environmentally embedded regulatory mechanisms, without requiring explicit internal representations.
(4) The framework integrates state‐dependent and process‐dependent dynamics, enabling comparative analysis of adaptive behaviour across phylogenetic and organisational scales.
(5) CDMD also provides empirically testable predictions and operational criteria, supporting experimental investigations into how historical and environmental constraints modulate adaptive decision‐making.

## CONFLICT OF INTEREST

The authors declare no conflicts of interest.

## Data Availability

Data sharing not applicable to this article as no datasets were generated or analysed during the current study.
